# In Situ Atomic‐Scale Study of Particle‐Mediated Nucleation and Growth in Amorphous Bismuth to Nanocrystal Phase Transformation

**DOI:** 10.1002/advs.201700992

**Published:** 2018-03-27

**Authors:** Junjie Li, Jiangchun Chen, Hua Wang, Na Chen, Zhongchang Wang, Lin Guo, Francis Leonard Deepak

**Affiliations:** ^1^ Department of Advanced Electron Microscopy Imaging and Spectroscopy International Iberian Nanotechnology Laboratory (INL) Avenida Mestre Jose Veiga 4715‐330 Braga Portugal; ^2^ School of Chemistry and Environment Beihang University Beijing 100191 China; ^3^ School of Materials Science and Engineering Tsinghua University Beijing 100084 China; ^4^ Department of Quantum Materials, Science and Technology International Iberian Nanotechnology Laboratory (INL) Avenida Mestre Jose Veiga 4715‐330 Braga Portugal

**Keywords:** aberration‐corrected transmission electron microscopy, in situ observations, kinetic processes, nucleation and growth, phase transitions

## Abstract

Understanding classical and nonclassical mechanisms of crystal nucleation and growth at the atomic scale is of great interest to scientists in many disciplines. However, fulfilling direct atomic‐scale observation still poses a significant challenge. Here, by taking a thin amorphous bismuth (Bi) metal nanosheet as a model system, direct atomic resolution of the crystal nucleation and growth initiated from an amorphous state of Bi metal under electron beam inside an aberration‐corrected transmission electron microscope is provided. It is shown that the crystal nucleation and growth in the phase transformation of Bi metal from amorphous to crystalline structure takes place via the particle‐mediated nonclassical mechanism instead of the classical atom‐mediated mechanism. The dimension of the smaller particles in two contacted nanoparticles and their mutual orientation relationship are critical to governing several coalescence pathways: total rearrangement pathway, grain boundary migration‐dominated pathway, and surface migration‐dominated pathway. Sequential strain analyses imply that migration of the grain boundary is driven by the strain difference in two Bi nanocrystals and the coalescence of nanocrystals is a defect reduction process. The findings may provide useful information to clarify the nanocrystal growth mechanisms of other materials on the atomic scale.

## 1 Introduction

Crystallization represents an important process in chemistry, materials science, and condensed‐matter physics.^1^, ^2^, ^3^, ^4^ Based on classical homogeneous nucleation and growth mechanism, a crystal nucleus is first generated by spontaneous random aggregation of species from liquid or solution, followed by atomic or molecular attachment to form a stable crystal structure, a usual process for crystallization.^5^, ^6^, ^7^ However, it is increasingly recognized that this mechanism is not suitable to all aspects of nucleation and growth process.^8^, ^9^, ^10^, ^11^ Recently, a prenucleation stage has been proposed and validated experimentally. In light of this new theory, an amorphous dense phase is first formed and subsequently reorders to form a thermodynamically stable crystal nucleus.^11^, ^12^, ^13^ Other nonclassical mechanisms are related to particle‐mediated growth and assembly mechanism, including the oriented attachment and mesocrystal formation process.^14^, ^15^, ^16^, ^17^

To clarify the classical and nonclassical crystallization mechanisms, several attempts have been devoted to image crystal growth at the nanometer or atomic scale by in situ liquid cell and cryo‐transmission electron microscopy (TEM).^11^, ^18^, ^19^, ^20^, ^21^, ^22^ For example, Alivisatos and co‐workers observed the growth trajectories of single colloidal platinum nanocrystal in solution and showed that the platinum nanocrystal can grow by either monomer attachment from solution or particle coalescence.^19^ Zheng and co‐workers reported the real‐time imaging of Pt_3_Fe nanorod growth in solution and identified a range of intricate processes in the growth of nanorods from nanoparticle building blocks.^21^ De Yoreo and co‐workers conducted real‐time observation of CaCO_3_ nucleation in solution and revealed that multiple nucleation pathways are simultaneously operative, including direct formation from solution and indirect formation via transformation of amorphous and crystalline precursors.^11^ These observations enrich the theory of crystal nucleation, providing direct evidence to nonclassical crystallization mechanisms at the nanoscale. However, due to the lack of direct experimental observations at the atomic scale as well as experimental intricacies in tackling such challenging systems, much confusion still exists regarding atomistic understanding of the classical and nonclassical nucleation and growth mechanisms.

To realize atomic‐scale observation of crystal growth in an electron microscope, Kim and co‐workers utilized conventional TEM to observe a nonclassical nucleation mechanism of multiphase transformation in amorphous to crystal phase transformation of LiFePO_4_ under heating.^8^ Nevertheless, due to the limit of spatial resolution of a conventional TEM, many critical details remain elusive, such as atomic‐scale nucleation and growth mechanism at initial stage, detailed coalescence process, role of coalescence during crystal growth, and migration of dislocation at grain boundary. With the advent of aberration‐corrected TEM (AC‐TEM), direct atom‐resolved imaging of even extremely small nanostructures is feasible.^23^, ^24^, ^25^, ^26^ For example, recently, Alivisatos and co‐workers used AC‐TEM to probe structural transformation dynamics of Cu_2_S nanoparticles at the atomic scale under electron beam irradiation.^27^

Here, we design and apply a thin amorphous bismuth (Bi) metal nanosheet as a model system to unveil nonclassical mechanism of crystal nucleation and growth in an amorphous to crystal phase transformation of Bi metal at the atomic scale under electron beam irradiation. We demonstrate that particle coalescence is crucial to the growth of initial stable crystal nucleus and subsequently single crystals. We offer definitive evidence to the cluster coalescence‐driven crystallization and identify the critical diameter of the formed stabilized crystal in the amorphous to crystalline phase transformation of Bi metal. Interestingly, the coalescence mode of nanoparticles can be controlled by the dimension of the smaller particle in the two contacted nanoparticles and their mutual orientation relationship. Further sequential geometrical phase analyses (GPAs) reveal a stress‐driven grain boundary migration.

## 2 Results and Discussion

Amorphous Bi nanosheets were synthesized using a simple ultrasonic route.^28^ Atomic force microscope image shows that the nanosheet has a thickness of ≈10 nm (Figure S1, Supporting Information). Low‐magnification TEM, selected area electron diffraction, high resolution TEM (HRTEM), and fast Fourier transformation (FFT) characterizations indicate that the nanosheet is of amorphous nature (Figure S2, Supporting Information). The corresponding energy‐dispersive X‐ray spectroscopy (EDS) mapping and spectrum verify their chemical composition (Figure S3, Supporting Information). It is well‐known that bulk Bi metal has a low melting point of 544.4 K and the melting temperature of its nanoparticles can be as low as room temperature due to the size effect.^29^ As such, the as‐obtained thin amorphous Bi nanosheet can act as an ideal model system to probe the crystal nucleation and growth mechanism.^30^, ^31^, ^32^, ^33^, ^34^

**Figure 1:**
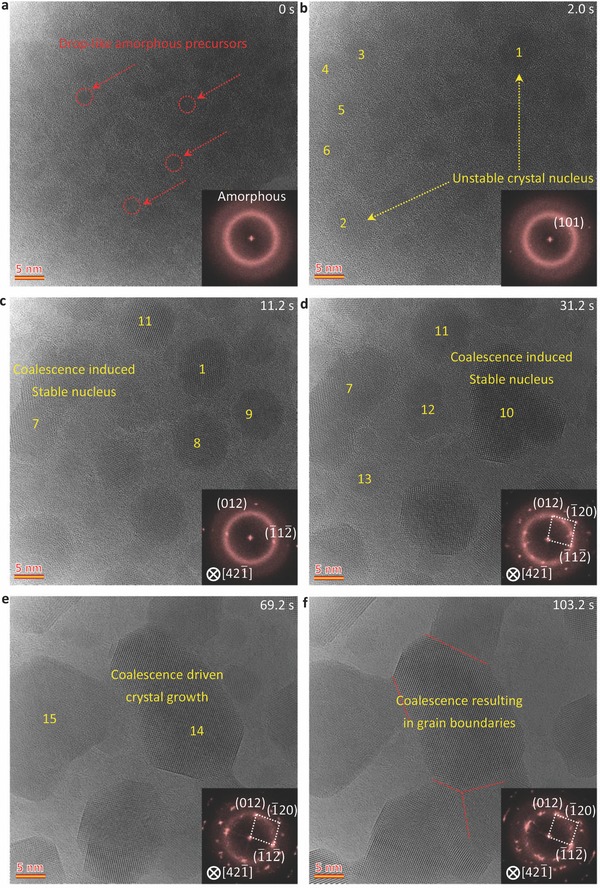
Nanoparticle‐mediated crystal nucleation and growth in amorphous Bi to crystal phase transformation. a–d) HRTEM images showing the nanoparticles coalescence‐mediated nucleation of nanocrystal. e,f) HRTEM images showing the nanoparticles coalescence‐induced growth of nanocrystal and the formation of grain boundaries. Inset images are the corresponding FFT patterns. The electron dose is 18 000 e A^−2^ s^−1^.

**Figure** **1** shows typical sequential atomic‐scale TEM images and corresponding FFT, revealing the phase transformation from amorphous Bi to crystalline Bi under electron beam irradiation (electron dose of ≈18 000 e A^−2^ s^−1^) (see also Video S1 in the Supporting Information). Figures S4 and S5 (Supporting Information) show that the Bi nanocrystal under electron irradiation possesses a hexagonal structure (JCPDS Card No. 44‐1246). From Figure 1a, one can see that many ultrasmall drop‐like circular amorphous precursors are formed either in the interior or at the surface of the amorphous Bi at the initial prenucleation stage (marked by red arrows), as confirmed by the FFT image (inset of Figure 1a). Subsequently, the small precursors grow up and undergo coalescence in a very short time: nanoparticles 1 and 2 (marked in Figure 1b) clearly show a Bi lattice structure as confirmed by the corresponding FFT analysis. However, the newly formed nanocrystal still retains the circular morphology even when its structure fluctuates in‐between cluster and crystal. When its size reaches a critical value, *d*_c_ ≈10 nm, the nanoparticle eventually develops into a nanocrystal with a stable crystal structure. Subsequent to the formation of many such small unstable nanoparticles, the nucleation and growth mediated by nanoparticle coalescence take place rapidly (Figure 1b–e): the nanoparticles 3–6 merge to form nanocrystal 7 (Figure 1c); nanoparticles 1, 8, and 9 merge to form nanocrystal 10 (Figure 1d); nanoparticles 10 and 11 merge to form nanocrystal 14; and nanoparticles 7, 12, 13 merge to form nanocrystal 15 (Figure 1e). These processes can be attributed to the high migration speed and high density of nanoparticles in the rapid nucleation process.^35^, ^36^ Accompanied with the increasing growth of the nanocrystals, atomic migration and rearrangement tend to become more difficult resulting in bi‐ or trijunction grain boundaries (Figure 1f).

**Figure 2:**
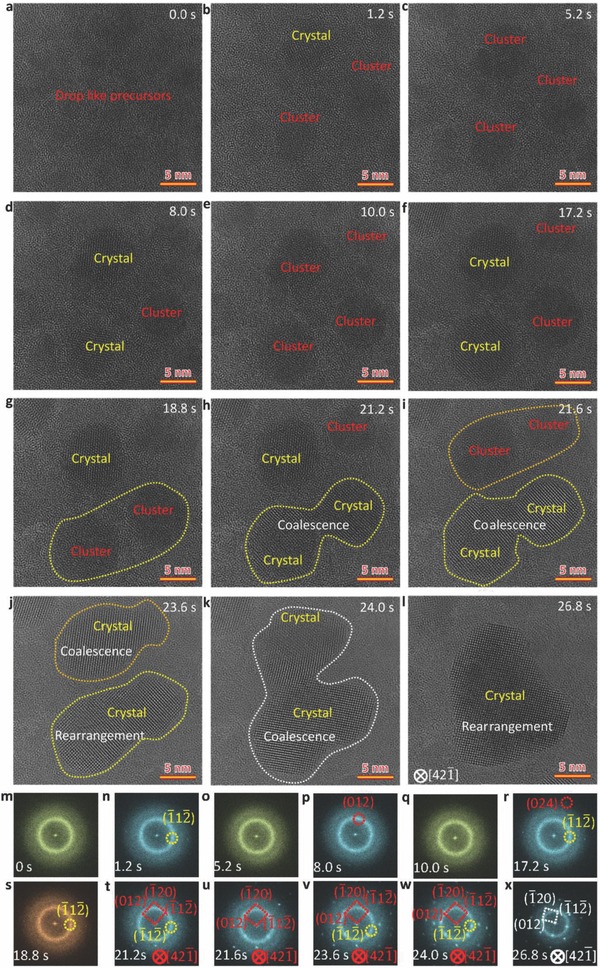
Detailed cluster coalescence‐induced Bi crystallization and growth in amorphous to crystal phase transformation. a–f) HRTEM images showing the structural fluctuations of unstable nucleus between cluster and nanocrystal. g–j) HRTEM images showing the cluster coalescence‐induced rapid crystallization. k,l) HRTEM images showing the coalescence of the two newly formed nanocrystals. m–x) Corresponding FFT patterns in (a–l) confirm the structural evolution in the phase transformation. The electron dose is 18 000 e A^−2^ s^−1^.

To shed light on the cluster coalescence‐induced crystallization, we show in **Figure** **2** enlarged sequential HRTEM images of nanoparticle coalescence (yellow area in Video S1 in the Supporting Information). The ultrasmall drop‐like amorphous precursor (≈3 nm) (Figure 2a) first grows to form an intermediate phase fluctuating between cluster and crystal (Figure 2b–i). Thereafter, the unstable nanoparticles contact with each other, undergoing a rapid coalescence to form a single crystal structure (Figure 2h–j), followed by a growth and rearrangement dominated by nanocrystal coalescence (Figure 2k,l). The corresponding sequential FFT analyses (Figure 2m–x) confirm the entire transformation sequence. To reveal clearly the coalescence of two clusters, we focus on the clusters with a size of ≈8 nm in Figure 2g (image area enlarged in Figure S6 in the Supporting Information). One can see that a neck‐like structure is formed after initial contact, which grows up in a very short time to form a single crystal. The cluster coalescence‐driven crystallization in phase transformation is related to the change in size and surface energy. When two nanoparticles contact, reduction of surface energy and bonding rearrangement may result in increase in local temperature, promoting atomic rearrangement, which benefits the formation of a nanocrystal with a larger size, stabilizing the crystal structure.^26^ Meanwhile, the rapid atomic rearrangement helps the newly formed nanocrystal to reach a faceted equilibrium shape within a short time after the coalescence.

**Figure 3:**
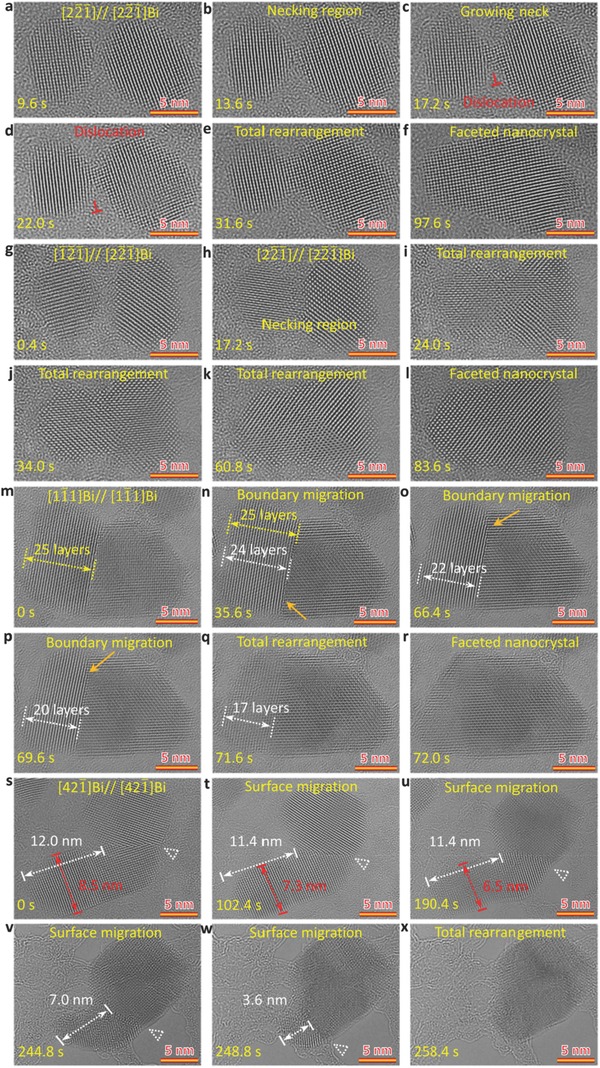
Sequential HRTEM images showing detailed size and orientation relationship‐dependent coalescence pathways. a–f) HRTEM images showing the rapid integral rearrangement pathway in two small nanocrystals with same orientations before contact. g–l) HRTEM images showing the rapid integral rearrangement pathway in two small nanocrystals with different orientations before contact. m–r) HRTEM images showing the grain boundary migration‐dominated pathway in two large nanocrystals with a rotation angle of 20°. s–x) HRTEM images showing the surface migration‐dominated pathway in two large nanocrystals with a rotation angle of 40°. The electron dose is 18 000 e A^−2^ s^−1^.

To gain insight into the influence of particle size and orientation on the coalescence mechanisms in the amorphous to crystalline phase transformation, we investigate the coalescence of nanocrystals with different sizes and orientations, as shown in **Figure** **3**. Figure 3a–f shows the coalescence dynamics of two small nanocrystals with a size of ≈8.5 and ≈10.5 nm, viewed along the [2$\bar{2}\bar{1}$] direction before coming into contact (see also Video S2 in the Supporting Information). Based on the FFT analyses (see also Figures S7a–f and S8 in the Supporting Information), the rotation angle between the two nanocrystals alters from 11° before contact to 0° after contact. It is worth noting that the main atomic rearrangement takes place in the smaller nanocrystal. Figure 3g–l shows the coalescence process of two small nanocrystals with a size of ≈8.0 and ≈10.1 nm, viewed along different orientations of [$\bar{1}\bar{2}\bar{1}$] and [2$\bar{2}\bar{1}$] before coming into contact (see also Video S3 in the Supporting Information), as also confirmed by FFT analyses (Figure S7g–l, Supporting Information). After coming into contact (Figure 3h), a grain boundary with an angle of 15° is observed (the angle is confirmed by FFT in Figure S9 in the Supporting Information), but disappears in ≈7 s through a rapid atomic rearrangement in the smaller nanocrystal by rotating to the same orientation as the bigger one. That is, when the size *d*_s_ of smaller nanoparticle in a pair of particles is smaller than the critical size *d*_c_, coalescence of nanoparticles shows a pathway of rotation and rearrangement of the smaller particle. In addition, the orientation relationship between the two nanoparticles has negligible influence on the entire coalescence process. However, the orientation of the big nanocrystal often dominates the final orientation of the newly formed nanocrystal after coalescence.^37^, ^38^, ^39^ In contrast, when the size of smaller nanoparticle is close to or larger than the critical size *d*_c_, the coalescence becomes more dependent on the orientation (Figure 3m–x). Compared to the rapid atomic migration and rearrangement for the small particles (particle size smaller than critical size *d*_c_), the slow atomic migration and rearrangement rates are observed in the big nanocrystals, resulting in the formation of relatively stable grain boundary after contact of the two particles.^17^, ^39^, ^40^

Figure 3m–r shows coalescence of two nanocrystals with a size of ≈13 nm (larger than *d*_c_), leading to the formation of a small high‐angle grain boundary with a rotation angle of 20° (see also Video S4 in the Supporting Information), as also confirmed by the corresponding FFT analyses (Figures S7m–r and S10, Supporting Information). The dynamic process confirms that the coalescence pathway is dominated by the grain boundary migration in the two nanocrystals with a small high‐angle grain boundary. Figure 3m–q clearly shows the grain boundary migration from right to left nanocrystal, which is accompanied with the increase of size in the right nanocrystal and the layer reduction of left nanocrystal from 25 to 17 layers. Interestingly, when the width of the left nanocrystal is smaller than ≈5 nm (17 layers) (Figure 3q), the left smaller nanocrystal undergoes a rapid atomic rearrangement to the right bigger one with the same orientation, which is similar to the observation in Figure 2g–l.

In contrast to the grain boundary migration‐dominated coalescence pathway for the two nanocrystals with a small high‐angle grain boundary, the two nanocrystals with a large high‐angle grain boundary show a surface migration‐dominated coalescence pathway, as shown in Figure 3s–x (Video S5, Supporting Information). The two nanocrystals form a large high‐angle grain boundary with a rotation angle of ≈40° (Figure 3s), as confirmed by the corresponding FFT analyses (Figures S7s and S11, Supporting Information), which is different from the small high‐angle grain boundary (Figure 3m–r). In this case, the position of the grain boundary (labeled by white triangles) remains stable (Figure 3s–v). However, the size of the bottom smaller nanocrystal reduces from ≈12.0 to ≈3.6 nm in length and from ≈8.5 to ≈5.0 nm in width (Figure 3s–w). This implies that the coalescence of two nanocrystals with a large rotation angle takes a surface migration‐driven coalescence pathway.^41^, ^42^, ^43^ The remaining smaller bottom nanocrystal shows a rapid atomic rearrangement mechanism (Figure 3w,x) similar to the one in Figure 3g–l,q,r. It is worthy of mentioning that the surface migration‐dominated coalescence is slower than the grain boundary migration‐dominated coalescence because the carbon support is damaged by electron beam as a result of long irradiation time (Figure 3v–x).^44^, ^45^

**Figure 4:**
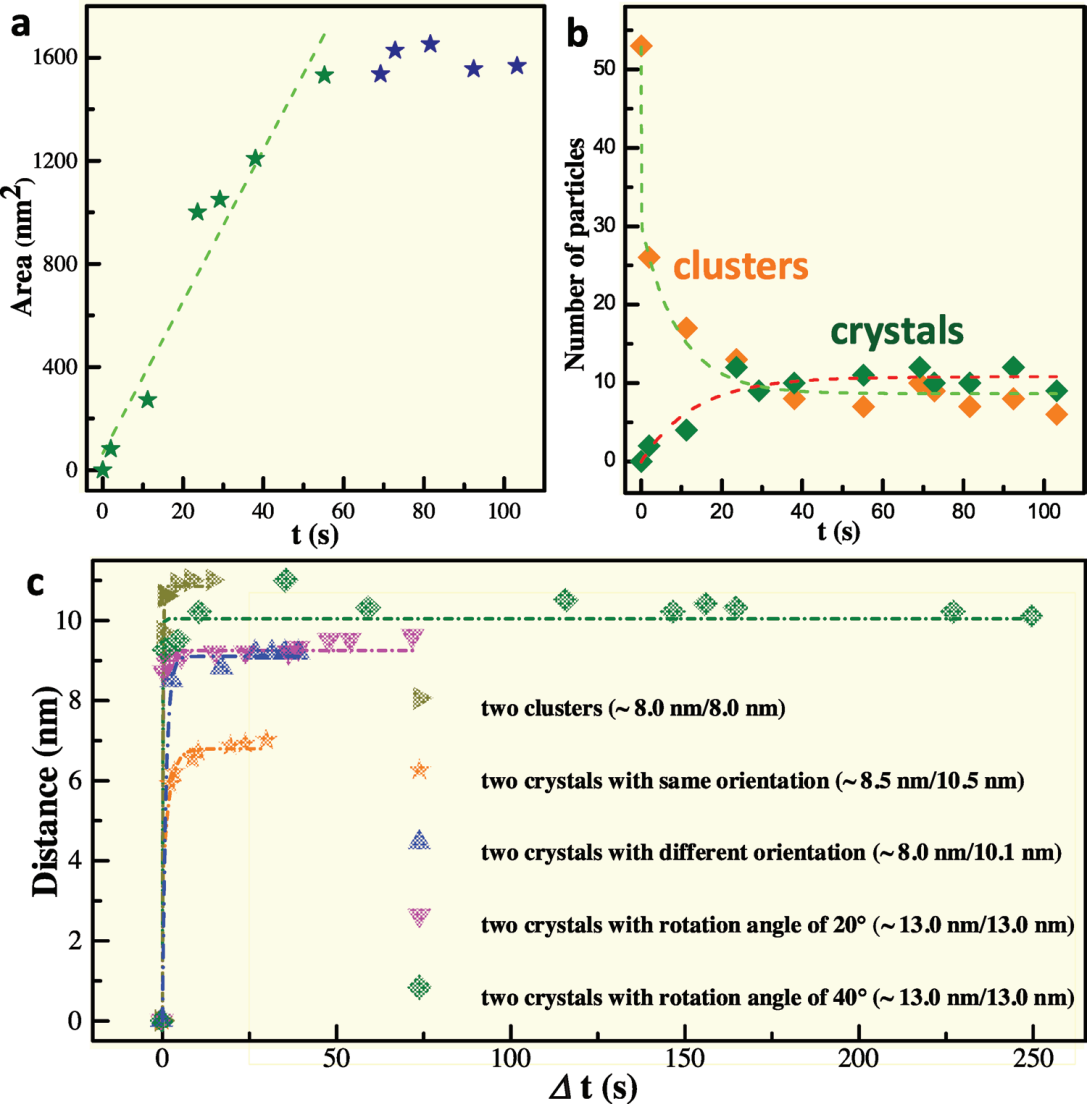
Crystalline and coalescence dynamic analyses in the phase transformation. a,b) The fluctuation of crystalline area, number of nanocrystal, and drop‐like nanoparticles with temporal evolution obtained from Video S1 (Supporting Information). c) Nonlinear coalescence dynamics processes of different nanoparticles in Figure 3 and Figure S6 (Supporting Information) and from Videos S1–S5 (Supporting Information), indicating that as the size of the smaller nanoparticles in the pair of particles is smaller than *d*_c_, the size imposes an important effect on the coalescence dynamics, while for the size of the smaller nanoparticles which is larger than *d*_c_, orientation relationship of two nanocrystals after contact imposes an important effect on the coalescence pathways and coalescence dynamics.

To further probe growth dynamic process in the amorphous to crystalline phase transformation, we plot crystallization area (*s*), number (*n*) of nanocrystal, and number (*m*) of drop‐like amorphous precursor as a function of crystallization time (Video S1, Supporting Information), as shown in **Figure** **4**a,b. The crystalline area shows a linear correlation with time at initial growth stage (labeled by green dotted line in Figure 4a). Once the amorphous Bi is fully consumed, the crystallization area steps into a relatively stable stage (Figure 4a), and the small fluctuation at this stage is ascribed to the surface energy‐induced atomic rearrangement. However, changes in the number of nanocrystals (*n*) and drop‐like amorphous precursors (*m*) show a nonlinear correlation with time, which is attributed to the coalescence‐driven rapid crystallization at the initial stage and also to the nanocrystal coalescence‐induced slow growth of bigger nanocrystals (Figure 4b).^46^

To uncover the coalescence dynamics for the nanoparticles with different sizes and orientations in the phase transformation, we plot separation between nanoparticle centers (*l*) as a function of width of smaller particles (*d)* and coalescence time (Δ*t* = *t*_c_ − *t*, where *t*_c_ is the time taken when two particles coalesce to form one particle) for the pairs of particles in Figure 3 and Figure S6 (Supporting Information). From Figure 4c, one can see a nonlinear correlation between *l* and Δ*t*, indicating that all coalescence processes are completed based on atomic rearrangement and migration. Comparing the three brown, orange, and blue curves, we can see that as the size of the smaller nanoparticles in the pair of particles is smaller than *d*_c_, their coalescence is finished in a short time after contact with each other. It implies that a similar coalescence pathway of small particle rotation and rearrangement is involved in these processes and the orientation relationship has a limited influence on the pathway. In contrast, when the size of the nanocrystal is larger than *d*_c_ (as shown by pink and green curves in Figure 4c), the orientation relationship of two nanocrystals after contact imposes an important effect on the coalescence pathway and the coalescence dynamics: surface migration pathway (the green curve, large high‐angle grain boundary in Figure 3s–x) takes more time than the grain boundary migration‐dominated pathway (the pink curve, small high‐angle grain boundary in Figure 3m–r).

**Figure 5:**
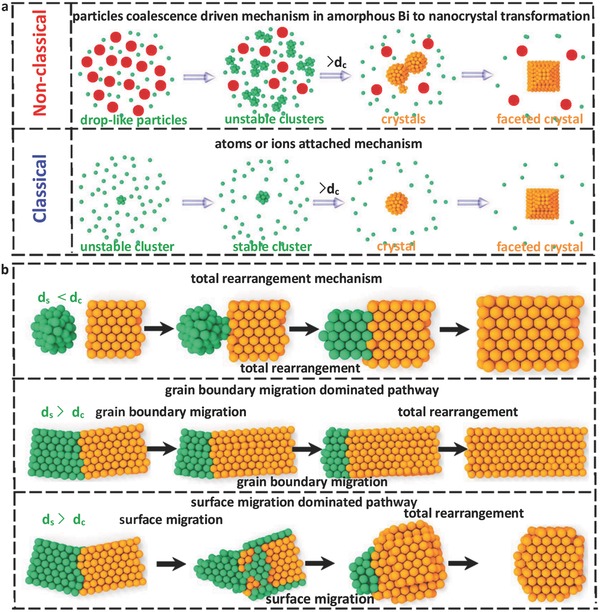
Schematics of nucleation and coalescence mechanisms. a) The schematics of the nonclassical mechanism of particle coalescence‐mediated nucleation and growth in amorphous Bi to nanocrystal and atom‐mediated classical mechanism. b) The schematics of the observed total rearrangement coalescence mechanism, grain boundary migration‐dominated pathway, and surface migration‐dominated pathway for the coalescence process.

**Figure** **5**a shows the schematic of the observed nonclassical mechanism of particle‐mediated nucleation and growth mechanism in the amorphous to crystalline phase transformation and classical atom‐mediated mechanism. Compared to the atom attachment‐mediated classical mechanism of crystal nucleation and growth, the nanoparticle coalescence plays an important role in forming stable nucleus, crystal, and faceted structures in the amorphous to crystalline phase transformation. At the initial stage, many unstable nuclei are formed in a short time and tend to migrate and undergo coalescence to form stable nuclei. Further migration and coalescence result in formation of a crystal. Subsequently, the coalescence of big nanocrystals generates stable grain boundaries after contact, leading to a polycrystalline nature. Figure 5b sketches the observed rearrangement and coalescence mechanism (*d*_s_ < *d*_c_), the grain boundary migration‐dominated (*d*_s_ > *d*_c_ and a small tilt angle after contact) pathway, and surface migration‐dominated (*d*_s_ > *d*_c_ and a large tilt angle after contact) pathway for the coalescence process.

**Figure 6:**
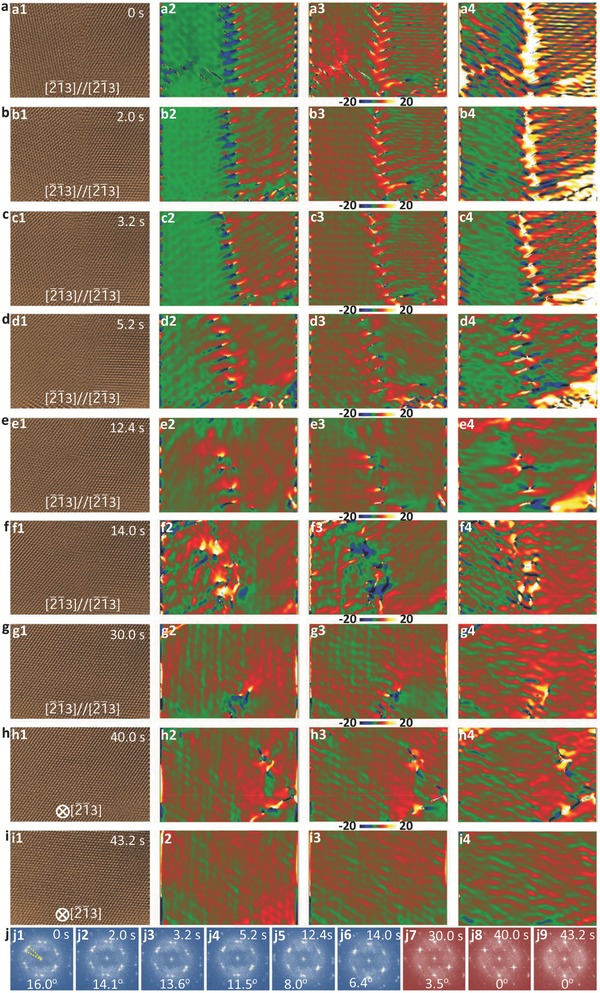
Sequential HRTEM and strain distribution images showing the migration of dislocation and grain boundary driven by strain in contacted nanocrystal. a–i) HRTEM images and the corresponding expansion (*ε_xx_*), shear components (*ε_xy_*), and contraction (*ε_xy_*) of the strain, showing the migration of dislocation and grain boundary during coalescence of nanocrystal. j) Corresponding FFT patterns confirming the linear change of rotation angle between the two nanocrystals with temporal evolution. The electron dose is 18 000 e A^−2^ s^−1^.

To further shed light on the migration of dislocation in the coalescence process, we show an atomic‐scale sequential HRTEM image of dislocation arrays at a grain boundary with a rotation angle of ≈16° viewed along [$\bar{2}\bar{1}$3] direction and the corresponding sequential strain tensor analyses in **Figure** **6**a–i (see also Video S6 in the Supporting Information). The strain tensor map of the corresponding HRTEM images is obtained using the GPA, where *ε_xx_*, *ε_xy_*, and *ε_xy_* are the expansion, shear components, and contraction of strain. From the initial HRTEM image (Figure 6a1) and strain distribution maps (Figure 6a2–a4), one can clearly notice the formed dislocation arrays at grain boundary and the different strain distributions on both sides of the grain boundary. Under electron irradiation, the atomic rearrangement takes place in the grain boundary, resulting in the migration and reduction of dislocations, which forms a new grain boundary (Figure 6a–f). Interestingly, accompanied with the migration of grain boundary, the expansion, shear components, and contraction of the strain are changed from the initial asymmetric distribution in both crystals to a more homogeneous distribution in both the nanocrystals, indicating that the migration direction of grain boundary is driven by the strain difference on both sides.^47^, ^48^, ^49^

To further understand the origin of strain evolution during the coalescence process, we investigate the change of rotation angle between the two nanocrystals with time employing the sequential FFT analyses (Figure 6j and Figures S12 and S13 (Supporting Information)). The rotation angle shows a linear inverse relationship with time, in accordance with the evolution of strain. When the rotation angle turns zero, all dislocations and grain boundaries are displaced out of this area, forming a single crystal structure with a uniformly distributed strain in this area. This emphasizes that the coalescence of nanocrystals is a defect (dislocation, grain boundary) reduction process.^37^, ^50^

## 3 Conclusion

The ability to perform in situ atomic‐resolution observation of dynamic process of crystallization and growth represents a significant step forward in understanding crystal nucleation and growth mechanisms at the atomic scale. We show that Bi crystal nucleation and growth in the transformation process from amorphous to crystalline phase take place via the nonclassical mechanism mediated by particle coalescence and that the coalescence pathway of two nanoparticles is governed by the dimension of the smaller particle and their orientation relationship. When the size of the smaller one in the two nanoparticles is less than the critical size, coalescence takes a thorough rearrangement and the rotation mechanism as well as the orientation has a negligible effect on the pathway. The coalescence of two crystals takes place via a grain boundary migration‐dominated pathway if the formed grain boundary is a small high‐angle grain boundary, while via a surface migration‐dominated pathway if a large high‐angle grain boundary is formed. Further sequential strain analyses imply that migration of a grain boundary is driven by strain difference in the two nanocrystals. These findings reveal the atomic‐scale dynamic information on the nonclassical particle‐mediated crystal nucleation and growth mechanism, which helps to advance our general understanding of dynamic process of phase transformation and nucleation.

## 4 Experimental Section

*Sample Preparation and Characterization*: The ultrasonic method was used to fabricate amorphous Bi nanosheets.^28^ In a typical synthesis process, a 100 mL ethanol solution containing bismuth powder of 300 mesh (350 mg) was sonicated for 5 h at 30 °C under an ultrahigh powder of 700 W. After that, the gray solution was concentrated at 1000 rpm for 30 min. The resultant transparent supernatant was used for the following experiments. The morphology and composition were confirmed by atomic force microscope, transmission electron microscopy, and corresponding energy‐dispersive X‐ray spectroscopy.

*In Situ TEM Observation and Chemical Analysis*: The obtained samples were dispersed in ethanol under ultrasonication for 30 min, and several drops of this dispersion were placed onto a holey carbon grid, which were subsequently dried for in situ TEM experiments. TEM imaging was conducted using a Titan Themis TEM equipped with both probe and image Cs correctors and a Super‐X EDS detector operated at 200 keV, which offered an unprecedented opportunity to probe structures with sub‐Ångström resolution. Series images were acquired using the Tecnai Imaging and Analysis software. The electron dose was tuned by manipulating spot size.

## Conflict of Interest

The authors declare no conflict of interest.

## Supporting information

Supplementary

Supplementary

Supplementary

Supplementary

Supplementary

Supplementary

Supplementary
