# Supplementary material for: In Situ Atomic‐Scale Study of Particle‐Mediated Nucleation and Growth in Amorphous Bismuth to Nanocrystal Phase Transformation
Source: Adv Sci (Weinh). 2018 Mar 27;5(6):1700992. doi: 10.1002/advs.201700992 (PMC6010897; doi:10.1002/advs.201700992)
Supplement: Supplementary file 1 — Supplementary [file ADVS-5-1700992-s002.pdf]

Copyright WILEY-VCH Verlag GmbH & Co. KGaA, 69469 Weinheim, Germany, 2018.

Supporting Information

for *Adv. Sci.,* DOI: 10.1002/advs.201700992

In Situ Atomic-Scale Study of Particle-Mediated Nucleation and Growth in Amorphous Bismuth to Nanocrystal Phase Transformation

Junjie Li, Jiangchun Chen, Hua Wang, Na Chen, Zhongchang Wang, Lin Guo, and Francis Leonard Deepak**

Supplementary Information for

In situ Atomic-Scale Study of Particle Mediated Nucleation and Growth in Amorphous Bi to Nanocrystal Phase Transformation

Junjie Li, Jiangchun, Chen, Hua Wang, Na Chen, Zhongchang Wang, Lin Guo and Francis Leonard Deepak**

Dr. J. Li and Dr. F. L. Deepak

Department of Advanced Electron Microscopy, Imaging and Spectroscopy

International Iberian Nanotechnology Laboratory (INL)

Avenida Mestre Jose Veiga, Braga 4715-330, Portugal

Email: leonard.francis@inl.int

Dr. Z. C. Wang

Department of Quantum Materials, Science and Technology

International Iberian Nanotechnology Laboratory (INL)

Avenida Mestre Jose Veiga, Braga 4715-330, Portugal

J. C. Chen, Dr. H. Wang, and Prof. L. Guo

School of Chemistry and Environment

Beihang University

Beijing 100191, China

Email: guolin@buaa.edu.cn

Prof. N. Chen

School of Materials Science and Engineering

Tsinghua University

Beijing 100084, China

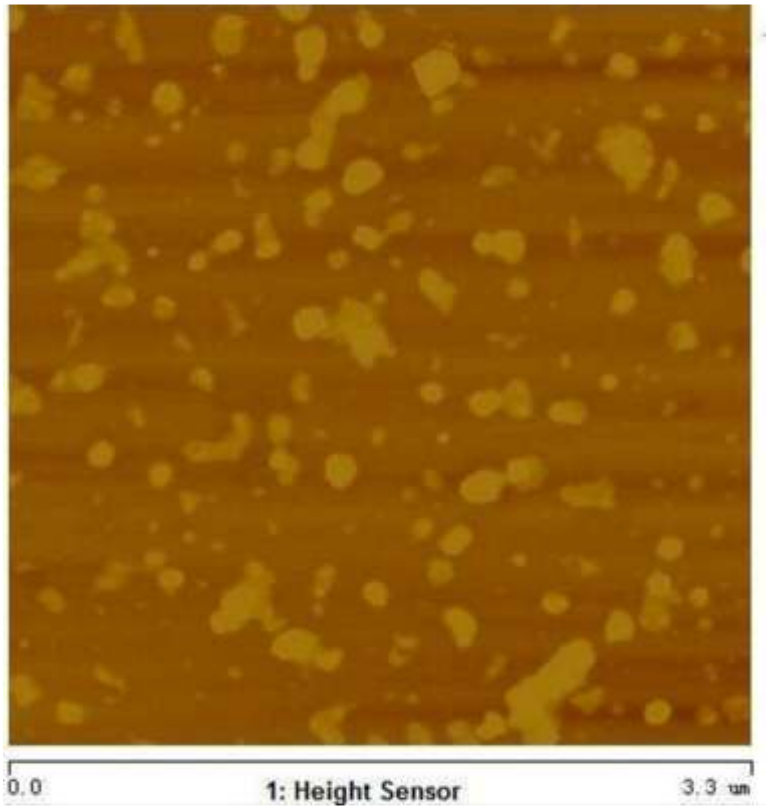

Supplementary Figure 1. Morphology of the sample. AFM image showing the thin Bi nanosheet structures.

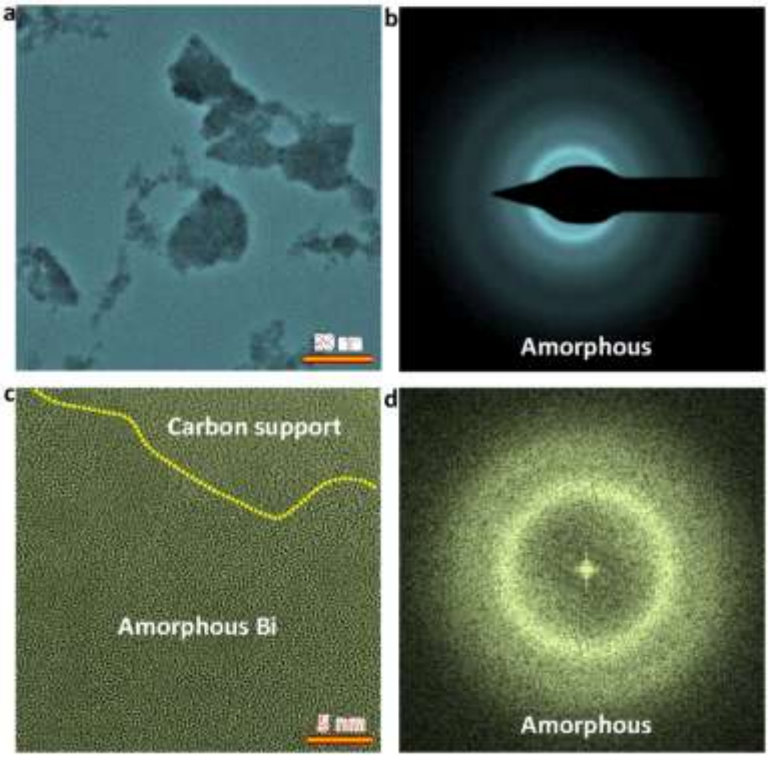

Supplementary Figure 2. Morphology of the sample. **a,b,** Low magnification TEM and corresponding selected area electron diffraction images for the obtained amorphous product. **c,d,** High magnification TEM and corresponding fast Fourier Transformation images for the obtained amorphous product.

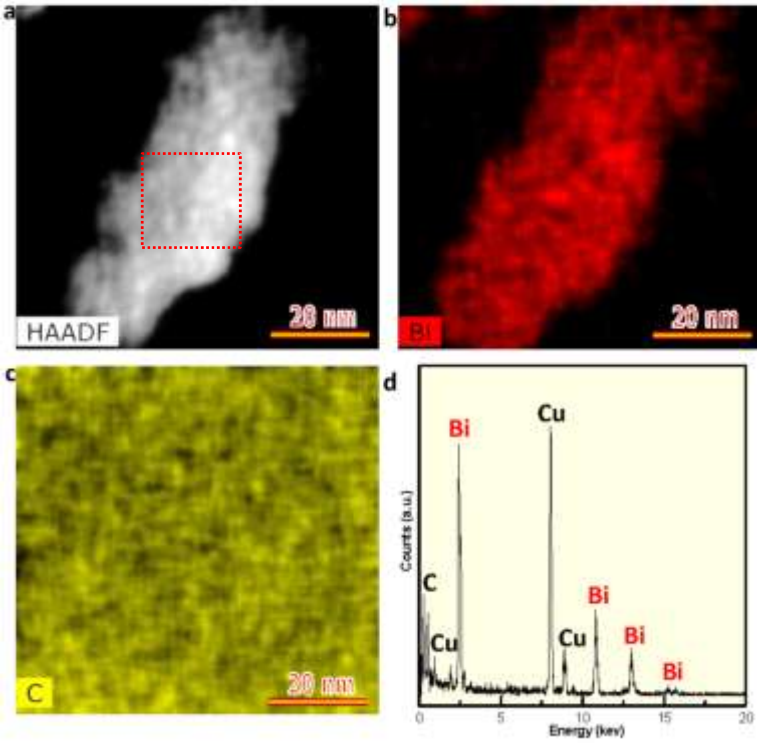

Supplementary Figure 3. Chemical mapping and spectrum of an amorphous Bi nanosheet. **a,** A HAADF STEM image of an amorphous Bi nanosheet on carbon support. **b–c,** Corresponding energy-dispersive x-ray spectroscopy (EDS) mapping of Bi (**b**) and C (**c**). **d,** EDS spectrum of the amorphous Bi nanosheet taken in the area marked by a red square in **a**.

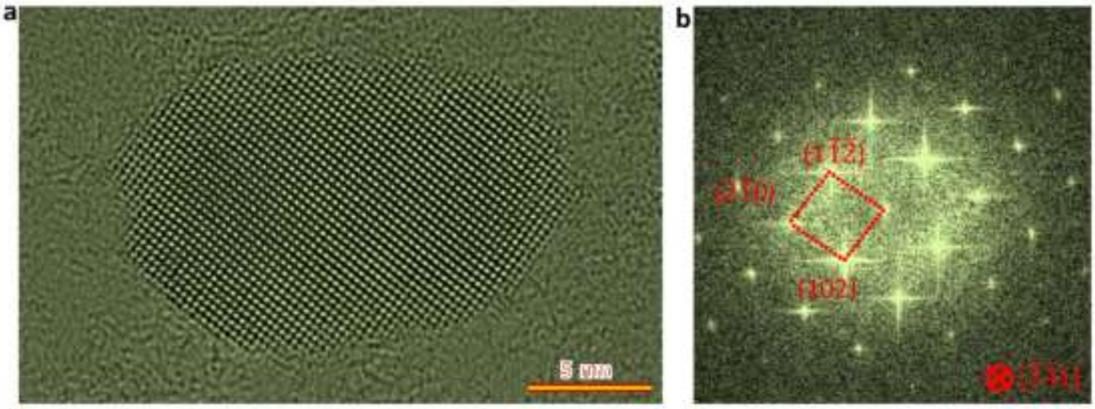

Supplementary Figure 4. Structure of the formed Bi nanocrystal under electron beam irradiation. **a,** High magnification TEM image of the obtained Bi nanocrystal. **b,** Corresponding fast Fourier Transformation image of the nanocrystal.

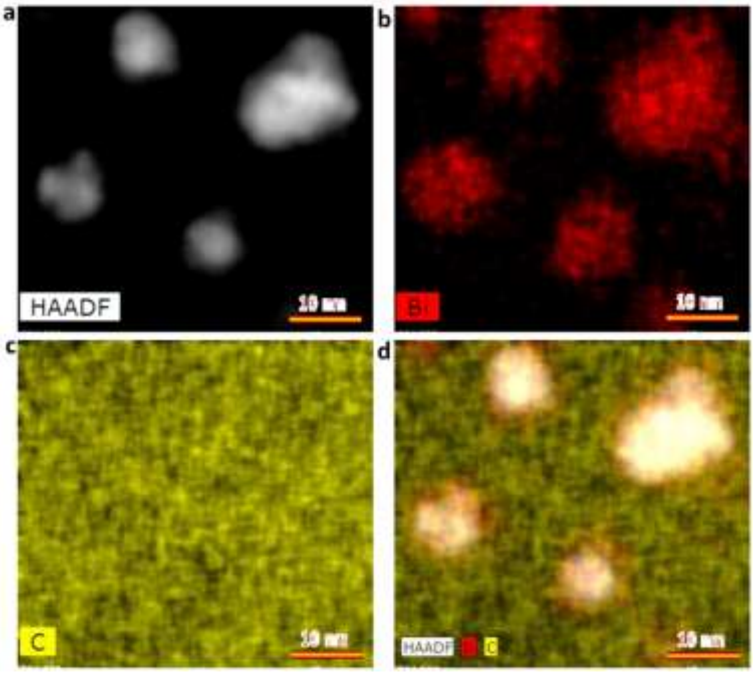

Supplementary Figure 5. Chemical mapping of the formed Bi nanocrystals under electron beam irradiation. **a,** A HAADF STEM image of Bi nanocrystals on carbon support. **b–d,** Corresponding energy-dispersive x-ray spectroscopy (EDS) mapping of Bi (**b**),C (**c**) and combined mapping (**d**).

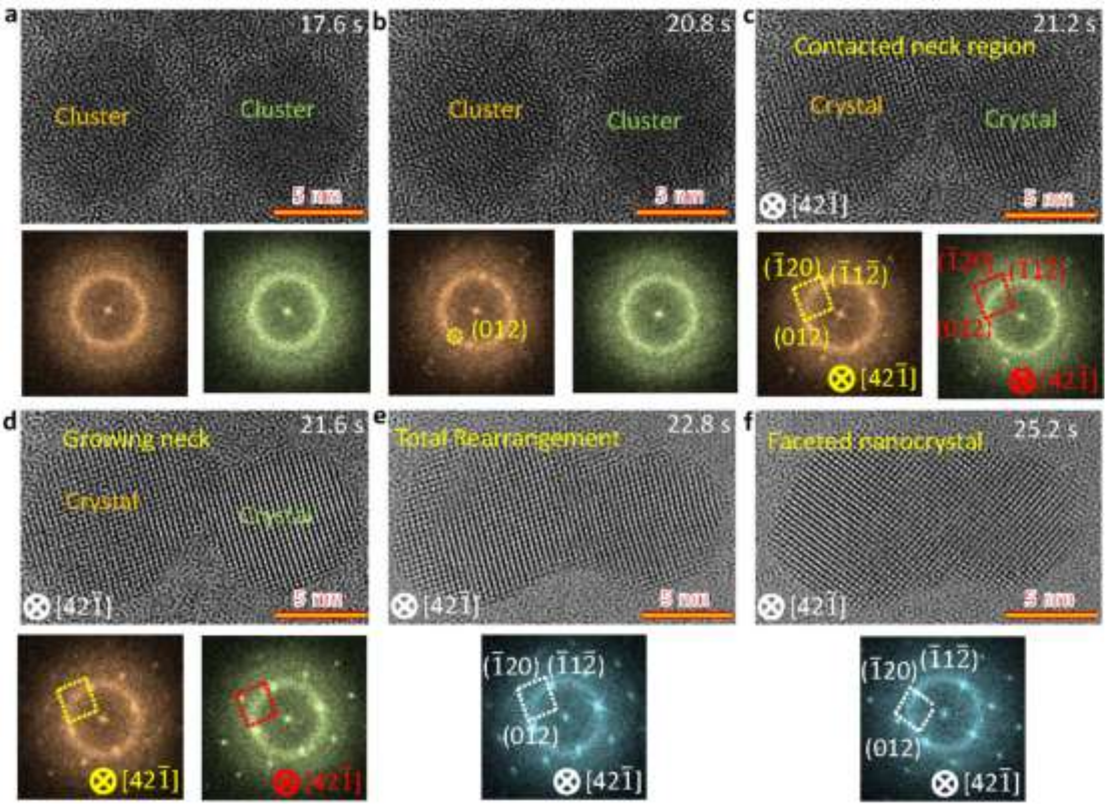

Supplementary Figure 6. Enlarged image of the cluster coalescence (Figure 2g) driven crystallization in phase transformation of amorphous Bi to crystalline Bi. **ab,** The growth and movement of both clusters. **c,** The formed crystal structure and the necking region after the two clusters contact. **d-f,** Atomic migration and rearrangement in the newly formed nanocrystal.

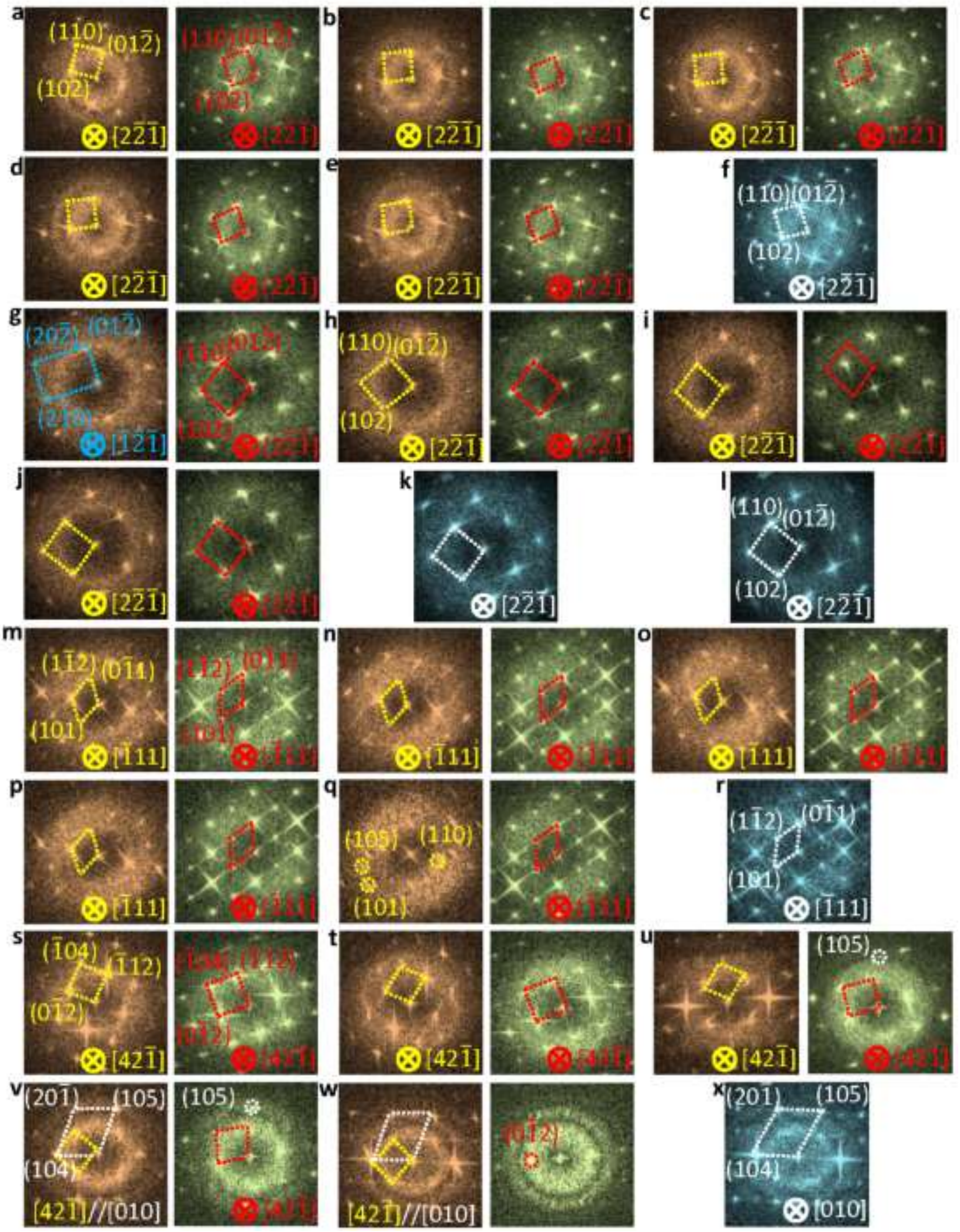

Supplementary Figure 7. FFT patterns show structural evolutions in the coalescence of the nanocrystal in Figure 3. **a-f,** FFT patterns obtained based upon the images in Fig. 3a-f. **g-l,** FFT patterns obtained based upon the images in Fig. 3g-l. **m-r,** FFT patterns obtained based upon the images in Fig. 3m-r.**s-x,** FFT patterns obtained based upon the images in Fig. 3s-x.

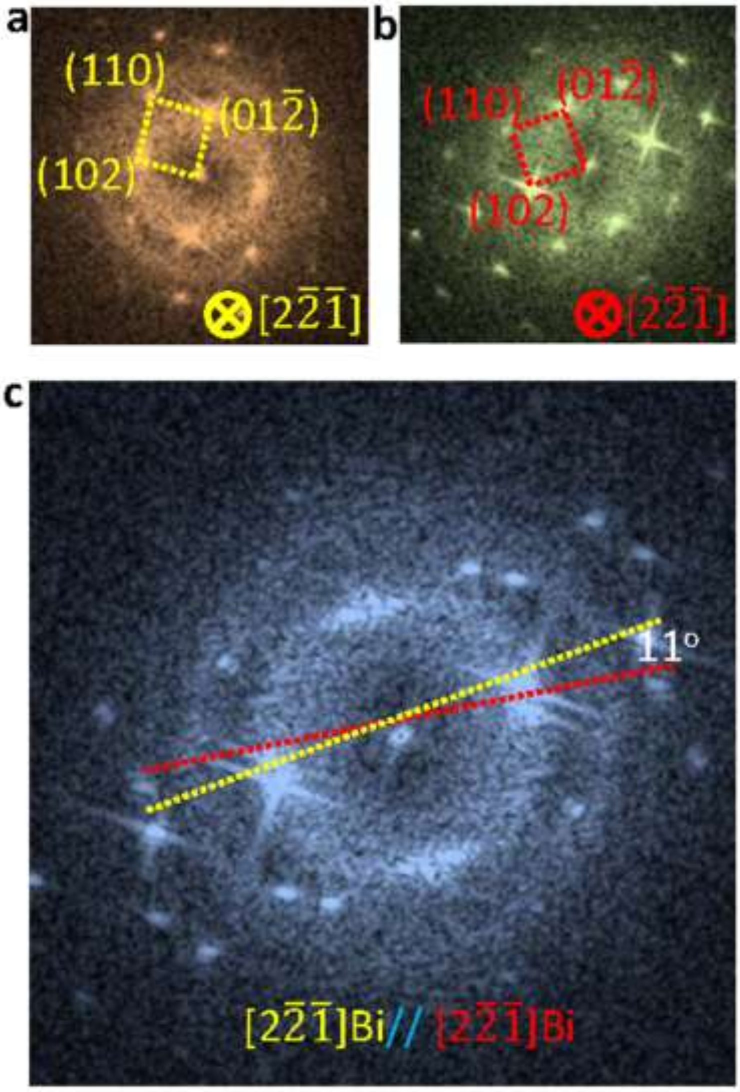

Supplementary Figure 8. FFT patterns obtained based upon the images in Figure 3a. **a,** FFT pattern of the left nanocrystal in Figure 3a. **b,** FFT pattern of the right nanocrystal in Figure 3a. **c,** FFT pattern of both the nanocrystals in Figure 3a which confirm the rotation angle of 11°.

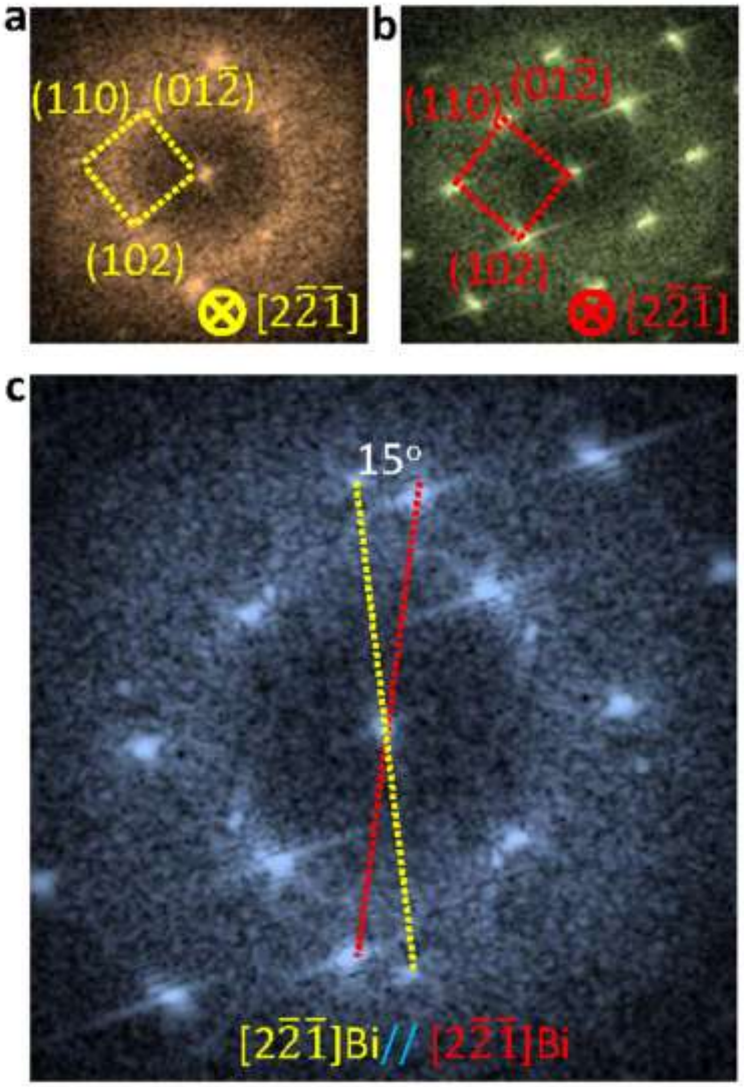

Supplementary Figure 9. FFT patterns obtained based upon the images in Figure 3h. **a,** FFT pattern of the left nanocrystal in Figure 3h. **b,** FFT pattern of the right nanocrystal in Figure 3h. **c,** FFT pattern of both the nanocrystals in Figure 3h confirming the rotation angle of 15°.

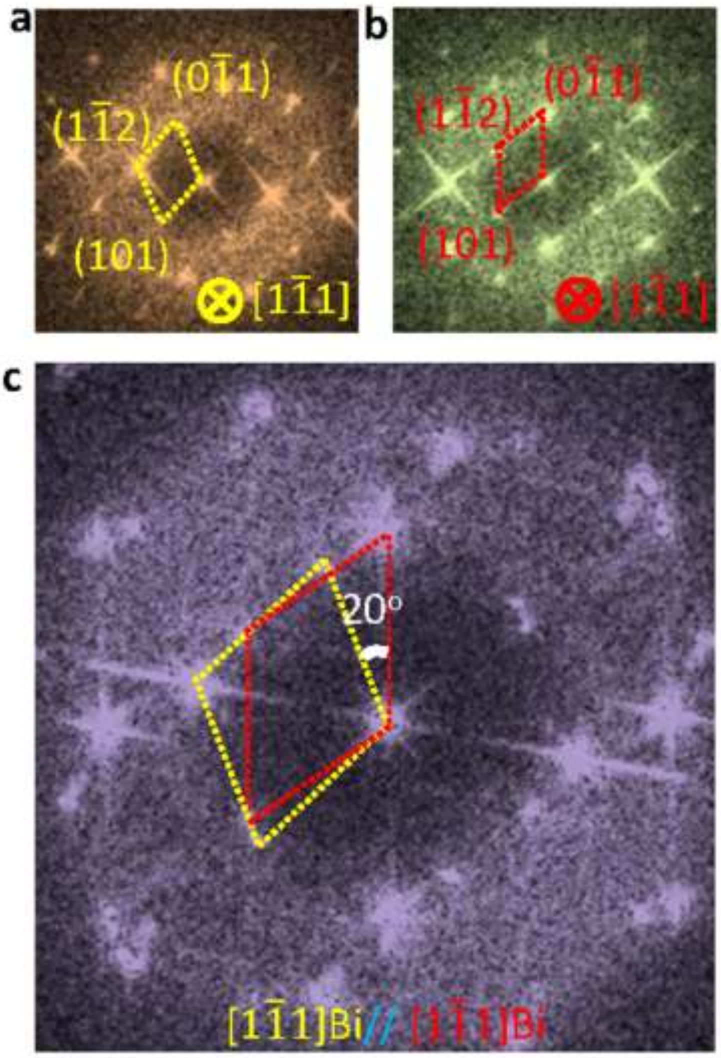

Supplementary Figure 10. FFT patterns obtained based upon the images in Figure 3m. **a,** FFT pattern of the left nanocrystal in Figure 3m. **b,** FFT pattern of the right nanocrystal in Figure 3m. **c,** FFT pattern of both the nanocrystals in Figure 3m confirming the rotation angle of 20°.

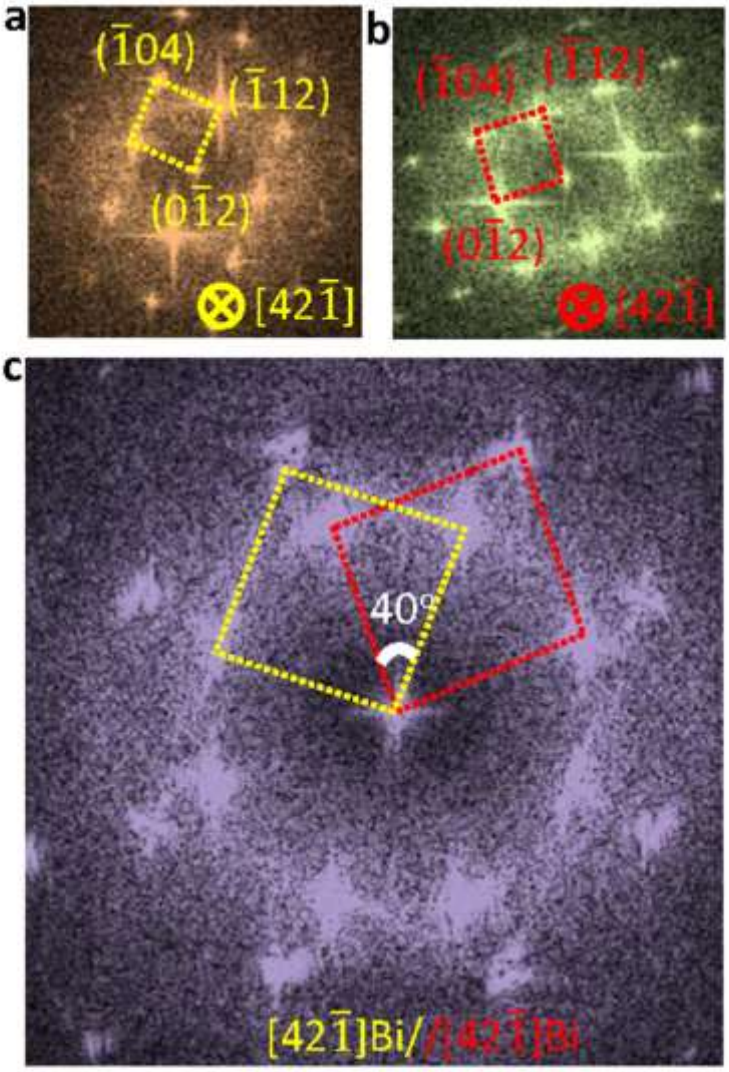

Supplementary Figure 11. FFT patterns obtained based upon the images in Figure 3s. **a,** FFT pattern of the top nanocrystal in Figure 3s. **b,** FFT pattern of the bottom nanocrystal in Figure 3s. **c,** FFT pattern of both the nanocrystals in Figure 3s confirming the rotation angle of 40°.

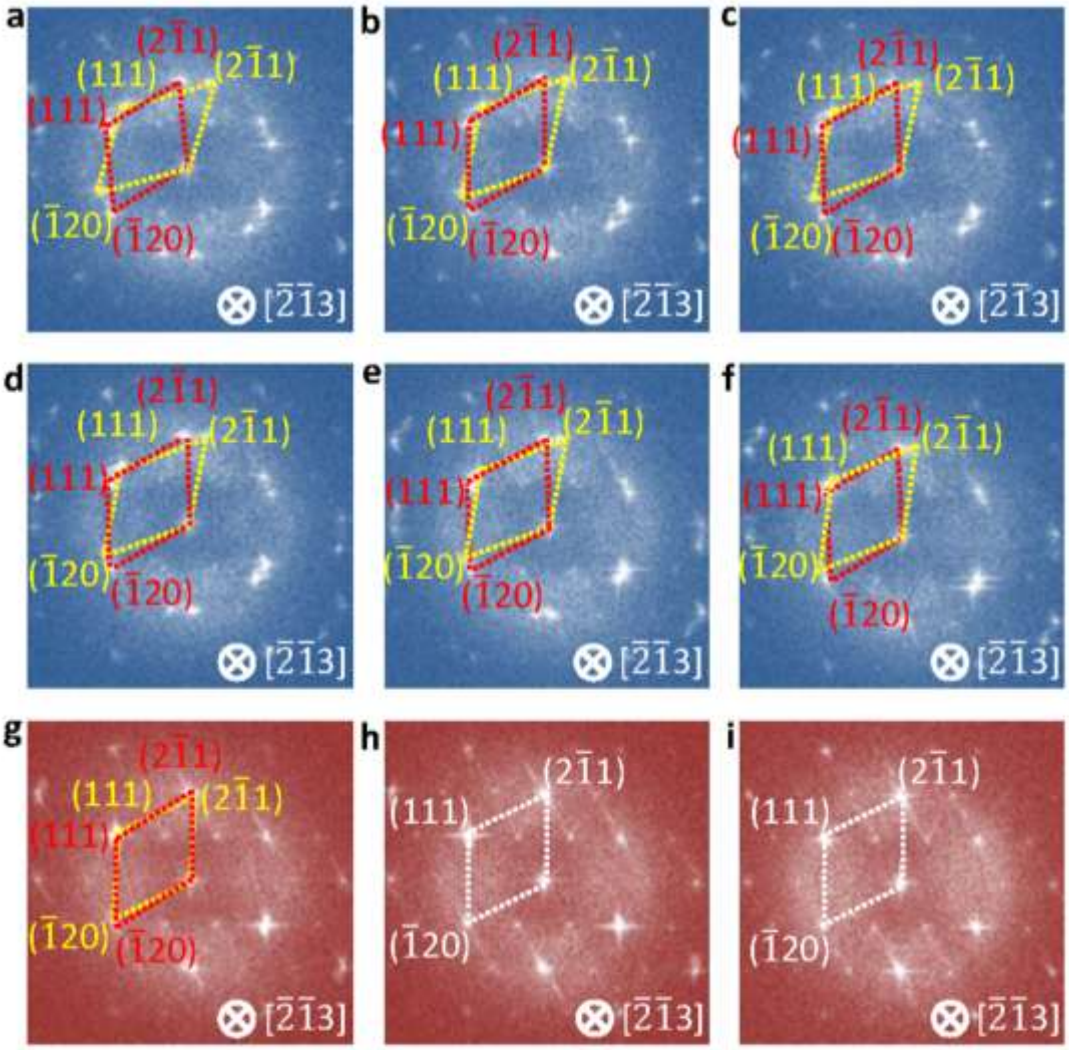

Supplementary Figure 12. Detailed FFT patterns obtained based upon the images in Figure 6. **a-g,** FFT patterns confirming the orientations and the rotation angle between nanocrystals. **h-i,** FFT patterns confirming the orientation of the new formed single crystal area.

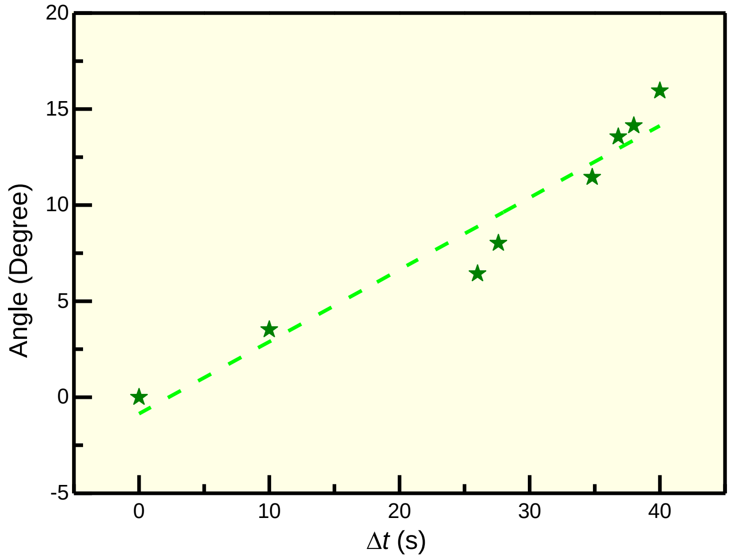

Supplementary Figure 13. The change of nanocrystal rotation angle along with time in Figure 6. The rotation angle shows a liner relationship with the time.

Videos Information

Video S1. Particle coalescence mediated nucleation and growth of Bi crystals and the formation of grain boundary under an electron dose of 18000 e/A^2 sec. The video plays at normal speed.

Video S2. The coalescence dynamics of two small nanocrystals with size of ~ 8.5 nm and ~ 10.5 nm, viewed along the same $[2\bar{2}\bar{1}]$ direction before coming into contact under an electron dose of 18000 e/A^2 sec. The video plays at normal speed.

Video S3. Stepped migration and coalescence of a nanocrystal with a droplet. The coalescence processes of two small nanocrystals with size of ~ 8.0 nm and ~ 10.1 nm viewed along different orientations of $[\bar{1}\bar{2}\bar{1}]$ and $[2\bar{2}\bar{1}]$ before coming into contact under an electron dose of 18000 e/A^2 sec. The video plays at normal speed.

Video S4. The coalescence of two nanocrystal both with sizes of ~13 nm and forming a high-angle grain boundary with a rotation angle of 20 degree under an electron dose of 18000 e/A^2 sec. The video plays at normal speed.

Video S5. The coalescence of two nanocrystal both with sizes of ~13 nm and forming a high-angle grain boundary with a rotation angle of 40 degree under an electron dose of 18000 e/A^2 sec. The video plays at 2X normal speed.

Video S6. The migration of dislocations and grain boundary during the coalescence process of two nanocrystals under an electron dose of 18000 e/A^2 sec. The video plays at a normal speed.
